# Diagnostik und Therapie lokaler Wundinfektionen

**DOI:** 10.1007/s00391-021-01984-7

**Published:** 2021-10-22

**Authors:** Joachim Dissemond

**Affiliations:** Klinik und Poliklinik für Dermatologie, Venerologie und Allergologie, Universitätsklinikum Essen, Hufelandstraße 55, 45122 Essen, Deutschland

**Keywords:** Bakterien, Chronische Wunden, TILI-Score, Wundheilung, Antiseptika, Bacteria, Chronic wounds, TILI score, Wound healing, Antiseptics

## Abstract

Lokale Wundinfektionen sind ein multidisziplinär relevantes Problem und sollten möglichst frühzeitig diagnostiziert und adäquat therapiert werden. Neben einer Stagnation der Wundheilung sind es insbesondere die drohende Weiterentwicklung zu systemischen Infektionen bis hin zur Sepsis, die gefürchtete, potenziell lebensbedrohliche Komplikationen darstellen. Einen besonders hohen und multidisziplinären Stellenwert hat diese Thematik bei der Behandlung von Patienten mit chronischen Wunden. Bislang gab es für die Diagnostik keine einheitlich akzeptierten Kriterien. Hier ermöglicht jetzt der neu entwickelte und validierte TILI-Score als Ergänzung zu den Vitalparametern und serologischen Werten die rasche Objektivierung lokaler Wundinfektionen. Ergänzend können mit dem W.A.R.-Score zudem Patienten mit erhöhtem Infektionsrisiko identifiziert werden. Durch diese einfach einzusetzenden Hilfsmittel kann die Indikation einer antiseptischen Wundtherapie individuell, schnell und unproblematisch eingeschätzt werden. Für viele Patienten mit chronischen Wunden ist dann Polihexanid das Wundantiseptikum der ersten Wahl. Die Indikation von Wundantiseptika sollte aber spätestens nach einer Therapiedauer von 14 Tagen kritisch überprüft werden.

## Abstract (en)

Local wound infections are a multidisciplinary challenge which should be diagnosed as early as possible and adequately treated. In addition to a stagnation of wound healing, it is in particular the threat of development into systemic infections and even sepsis that represent feared, potentially life-threatening complications. This topic has a particularly high and multidisciplinary significance in the treatment of patients with chronic wounds. Until now, there were no generally accepted criteria for the diagnostics. The newly developed and validated TILI score, as a supplement to vital signs and serological values, enables rapid objectification of local wound infections. In addition, the W.A.R. score can be used to identify patients with an increased risk of infections. With these easy to use tools, the indications for antiseptic wound treatment can be assessed individually, quickly and without problems. For many patients with chronic wounds, polihexanide is then the wound antiseptic of first choice. However, the indications for wound antiseptics should be critically reviewed after a treatment duration of 14 days at the latest.

Die möglichst frühzeitige Diagnostik und adäquate Therapie lokaler Wundinfektionen ist ein multidisziplinär relevantes Problem. Einen besonders hohen Stellenwert hat diese Thematik bei der Behandlung von Patienten mit chronischen Wunden [1, 2]. Das Risiko, eine chronische Wunde zu entwickeln, steigt mit zunehmendem Lebensalter deutlich an. Entsprechend der aktuellen Definition der Fachgesellschaft Initiative Chronische Wunden (ICW) e. V. werden Wunden als chronisch bezeichnet, wenn sie nach 8 Wochen nicht abgeheilt sind. Unabhängig von dieser zeitlich orientierten Definition gibt es Wunden, die von Beginn an als chronisch anzusehen sind, da ihre Behandlung eine Therapie der weiterhin bestehenden Ursache erfordert. Hierzu gehören beispielsweise das diabetische Fußulkus (DFU), Wunden bei peripherer arterieller Verschlusskrankheit (pAVK), Ulcus cruris venosum oder Dekubitus [3].

Lokale Wundinfektionen können zu erheblichen klinischen Problemen führen. Neben einer Stagnation der Wundheilung ist es insbesondere die drohende Weiterentwicklung zu systemischen Infektionen bis hin zu einer Sepsis, die gefürchtete, potenziell lebensbedrohliche Komplikationen darstellen.

## Bakteriennachweis

Die Diagnostik von Patienten mit chronischen Wunden kann entsprechend der ABCDE-Regel [4] strukturiert erfolgen (Tab. 1). Mit dem Buchstaben „B“ werden hier alle klinisch relevanten Maßnahmen der Bakteriendiagnostik beschrieben. Für den Nachweis von Bakterien in chronischen Wunden ist in der klinischen Routine meist ein bakteriologischer Abstrich der Wundoberfläche ausreichend ([5]; Tab. 2).

**Table 1:** 

A	Anamnese
B	Bakterien
C	Klinische Untersuchung
D	Durchblutung
E	Extras

**Table 2:** 

Bakteriologischer Abstrich *ohne* vorherige Wundsäuberung	Nachweis/Ausschluss multiresistenter Erreger (Screening)
Bakteriologischer Abstrich *mit* vorheriger Wundsäuberung	Auffinden kausaler Erreger bei klinisch relevanter Wundinfektion
	Besiedlung/Infektion mit Hefepilzen
Biopsie für die Erregerdiagnostik	Wundinfektion bei tieferen Wunden, diabetischem Fußulkus
	Fistelgewebe, wenn kein Fistelinhalt gewonnen werden kann
	Vermutete Erreger: Mykobakterien, Leishmanien, Aktinomyzeten, Nocardien, Schimmelpilze
	Wundinfektion ohne Erregernachweis im Abstrich

**Figure 1:**
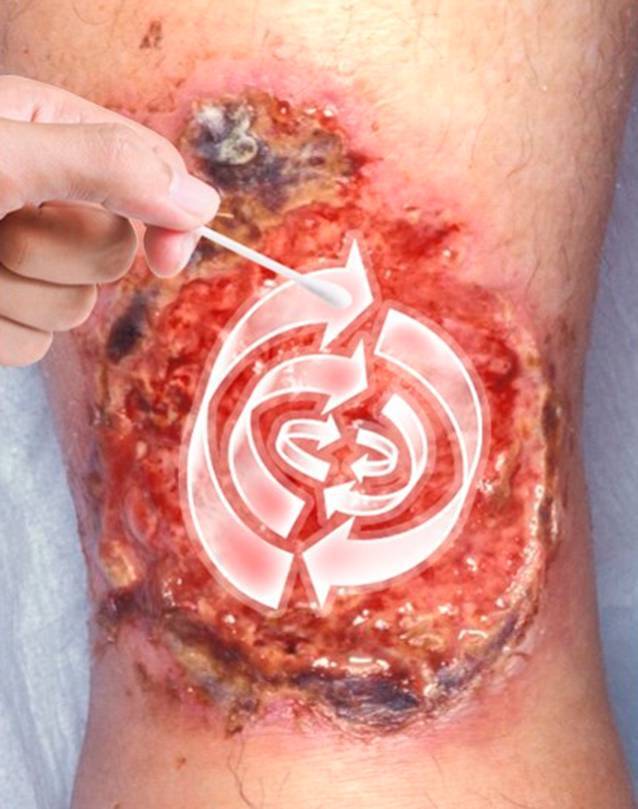

**Figure 2:**
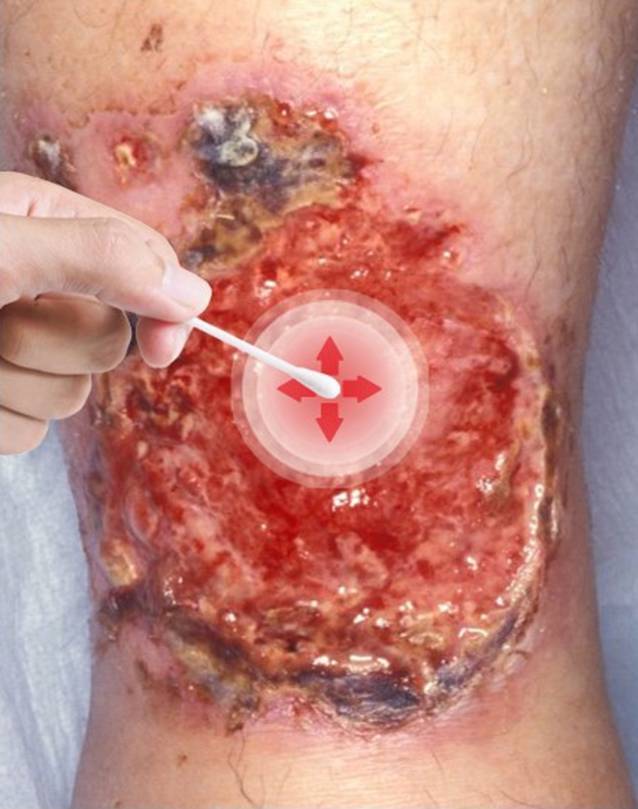

Wenn die Indikation für den Abstrich eine Screeninguntersuchung beispielsweise für den Nachweis von multiresistenten Erregern (MRE) ist, dann sollte zuvor keine Wundsäuberung erfolgen, um möglichst das gesamte Spektrum der Erreger nachweisen zu können. Die Durchführung entsprechend der Technik des Essener Kreisels [6] stellt hier eine validierte, leicht anwendbare Methode dar (Abb. 1). Bei klinischem Verdacht auf eine Wundinfektion sollte vor der Durchführung der bakteriologischen Diagnostik eine Wundsäuberung beispielsweise mit steriler physiologischer Kochsalz- oder Ringer-Lösung und sterilen Kompressen erfolgen, um klinisch nicht relevante Kontaminanten von der Oberfläche zu entfernen [5]. Hier wird die Durchführung eines bakteriologischen Abstrichs entsprechend der Levine-Technik in dem klinisch infiziert erscheinenden Areal empfohlen (Abb. 2). Eine Entnahme von Biopsien für die Erregerdiagnostik ist für die meisten Patienten mit chronischen Wunden nicht notwendig. Biopsien sollten allerdings bei Patienten mit Wundinfektionen und tieferen Ulzerationen, DFU, schweren Weichgewebeinfektionen, Fistelgewebe oder V. a. spezifische Erreger wie beispielsweise Mykobakterien, Leishmanien oder Schimmelpilze erfolgen [5].

## Stadien der Wundinfektion

Im angloamerikanischen Sprachraum wird oft der Begriff Infektionskontinuum [7] verwendet, um die verschiedenen Stadien von der harmlosen bakteriellen Kontamination bzw. Besiedelung bis zu einer potenziell gefährlichen, systemischen Infektion zu spezifizieren (Tab. 3). Hierbei werden mehrere aufeinanderfolgende klinische Zustände beschrieben [8, 9]. Die Einstufung als kritische Kolonisation wird allerdings zunehmend kontrovers diskutiert, da es hierfür keine gut objektivierbaren diagnostischen Kriterien gibt [10].

**Table 3:** 

Terminus	Merkmale
Kontamination	Mikroorganismen haben sich an die Wundoberfläche angelagert, vermehren sich jedoch nicht
Kolonisation	Mikroorganismen vermehren sich in der Wunde, es ist aber keine klinisch relevante Wirtsreaktion erkennbar
Kritische Kolonisation	Mikroorganismen vermehren sich in der Wunde ohne typische klinische Infektionszeichen. Es kommt aber u. a. zu stagnierender Wundheilung, pathologischer Granulation und Exsudationszunahme
Lokale Infektion	Immunologische Wirtsreaktion mit typischen klinischen Infektionszeichen (s. TILI-Score)
Systemische Infektion	Zu den lokalen Entzündungsreaktionszeichen kommen noch systemische immunologische Wirtsreaktionen wie z. B. Leukozytose, Anstieg des C‑reaktiven Proteins und Fieber

Wichtig ist es, bei der Abklärung lokaler Wundinfektionen eine systemische Infektion bzw. eine beginnende Sepsis nicht zu übersehen. Als systemic inflammatory response syndrome (SIRS) [11] werden systemische Entzündungsreaktionen des Körpers bezeichnet. Wird ein SIRS durch Infektionen hervorgerufen, spricht man von einer Sepsis. Eine Sepsis kann beispielsweise mit den Kriterien des quick Sepsis-related Organ Failure Assessment (qSOFA) Score [12] ermittelt werden. Hiermit wird das klinische Screening ohne Labortests rasch durchgeführt. Bei Verdacht auf Sepsis sind die systemische Gabe einer kalkulierten systemischen Antibiotikagabe und oft auch eine intensivmedizinische Betreuung indiziert [13].

## Therapeutischer Index für Lokale Infektionen

Durch eine Expertengruppe der ICW wurde 2019 der Score des Therapeutischen Index für Lokale Infektionen (TILI) für die Diagnostik lokaler Wundinfektionen entwickelt. Dieser TILI-Score wurde dann in mehrere Sprachen übersetzt und in einer multinationalen Studie validiert [14]. Daraufhin konnte eine aktualisierte Version 2.0 (Tab. 4) in Kooperation mit Wund-DACH, dem Dachverband der deutschsprachigen Wundheilungsgesellschaften, erstellt werden [15].

**Table 4:** 

Keine direkte Indikation	Direkte Indikation
Periläsionales Erythem	Nachweis potenziell pathogener Mikroorganismen^b^
Überwärmung	Chirurgische septische Wunde
Ödem, Verhärtung oder Schwellung	Freier Eiter
Spontaner Schmerz oder Druckschmerz^a^	
Stagnation der Wundheilung	
Anstieg und/oder Änderung der Farbe oder des Geruchs des Exsudats	

^a^Vorsicht bei Patienten mit Polyneuropathie oder bei Einnahme von Schmerzmitteln

^b^Dies kann in verschiedenen Ländern und Institutionen sehr unterschiedlich sein. Ein Beispiel ist der Nachweis von multiresistenten Bakterien wie Methicillin-resistentem *Staphylococcus aureus* (MRSA)

**Figure 3:**
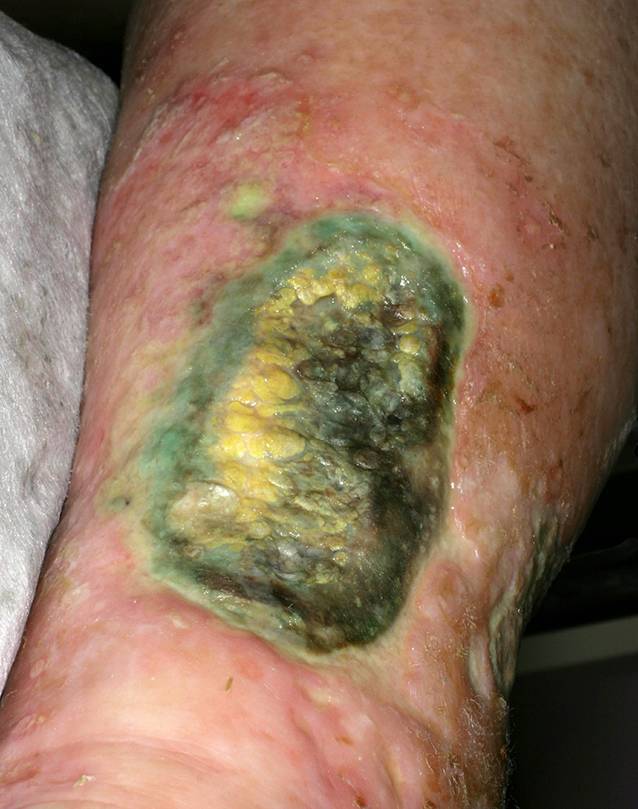

Für den TILI-Score wurden jedem der klassischen Entzündungszeichen die entsprechenden Symptome in der Wundheilung zugeordnet: Calor – Überwärmung; Dolor – spontaner Schmerz oder Druckschmerz; Tumor – Ödem, Verhärtung oder Schwellung; Rubor – periläsionales Erythem; Functio laesa – Stagnation der Wundheilung. Zusätzlich hat die Expertengruppe den Parameter Anstieg und/oder Änderung der Farbe oder des Geruchs des Exsudats ergänzt. Die hier genannten klinischen Symptome sind, einzeln betrachtet, wichtig, aber nicht beweisend für eine Infektion. Daher ergibt sich im TILI-Score erst dann eine Indikation für eine antiseptische Wundbehandlung, wenn mindestens 5 der 6 unspezifischen Symptome vorliegen ([14]; Abb. 3).

Darüber hinaus gibt es aber auch einzelne Aspekte, die eine antiseptische Wundtherapie rechtfertigen. Hier wurden der Nachweis potenziell pathogener Mikroorganismen, die chirurgische septische Wunde und freier Eiter als direkte Parameter identifiziert (Tab. 4).

## Wound-At-Risk (W.A.R.) Score

Im klinischen Alltag gestaltet es sich oft schwierig, Patienten zu identifizieren, bei denen noch keine Wundinfektion vorliegt, aber ein erhöhtes Risiko für die Ausbildung einer Wundinfektion besteht. Diese Wunden werden auch als Wound-at-risk (W.A.R.) bezeichnet. Bei diesen Patienten kann es im Gegensatz zu anderen Patienten sinnvoll sein, eine antimikrobielle Wundtherapie frühzeitiger und ggf. sogar langfristig durchzuführen. Daher hat eine interdisziplinäre Expertengruppe den W.A.R.-Score erarbeiten, mit dem das Infektionsrisiko anhand verschiedener endogener und exogener Faktoren ermittelt werden kann ([16]; Tab. 5). Auch dieser W.A.R.-Score kann schnell und unproblematisch im klinischen Alltag ermittelt werden [17].

**Table 5:** 

*1 Risikopunkt*
Erworbene immunsuppressive Erkrankung, z. B. Diabetes mellitus
Erworbener Immundefekt durch medikamentöse Therapie wie Ciclosporin, Methotrexat, Glukokortikoide, Antikörper
Erkrankungen mit soliden Tumoren
Hämatologische Systemerkrankung
Postchirurgische Wundheilungsstörung, welche zu (ungeplanter) Sekundärheilung führt
Durch Lokalisation besonders keimbelastete Wunden, z. B. Perineum, Genitale
Problematische hygienische Bedingungen durch soziales oder berufliches Umfeld, z. B. Landwirte, Lkw-Fahrer
Lebensalter ≥ 80 Jahre
Geringeres Lebensalter des Patienten, z. B. Frühgeborene, Babys, Kleinkinder
Bestehensdauer der Wunde > 1 Jahr
Wundgröße ≥ 10 cm^2^
Chronische Wunden aller Kausalitäten mit einer Tiefe > 1,5 cm
Stationärer Langzeitaufenthalt des Patienten > 3 Wochen
*2 Risikopunkte*
Schwere erworbene Immundefekte, z. B. HIV-Infektion
Stark verschmutze Akutwunden
Biss‑, Stich- und Schusswunden zwischen 1,5 und 3,5 cm Tiefe
*3 Risikopunkte*
Verbrennungswunden mit Beteiligung > 15 % der Körperoberfläche
Wunden, welche eine direkte Verbindung zu Organen oder Funktionsstrukturen aufweisen, z. B. auch Gelenke bzw. körperfremdes Material enthalten
Schwerste, angeborene Immundefekte wie beispielsweise Agammaglobulinämie, schwere kombinierte Immundefekte (SCID)
Biss‑, Stich- und Schusswunden > 3,5 cm Tiefe

Bei einem Gesamtwert ≥ 3 im W.A.R-Score wird eine antimikrobielle Wundbehandlung empfohlen

## Moderne Wundtherapie

Die Planung einer modernen Behandlung chronischer Wunden kann sich heute an dem MOIST-Konzept orientieren [18]. Bei diesem Akronym werden die verschiedenen Wundbehandlungsoptionen in mehrere Kategorien unterteilt (Tab. 6).

**Table 6:** 

M	„Moisture balance“ = Exsudatmanagement
O	„Oxygen balance“ = Sauerstoffbalance
I	„Infection control“ = Infektionskontrolle
S	„Support“ = Unterstützung des Heilungsprozesses
T	„Tissue management“ = Gewebemanagement

Mit dem Buchstaben T („tissue management“) wird das sog. Gewebemanagement beschrieben. Wichtige Bestandteile sind hierbei die Wundreinigung und das Débridement. Für die Wundreinigung sollte sterile Ringer- oder physiologische Kochsalzlösung verwendet werden. Nach Anbruch ist die Sterilität jedoch nicht mehr gewährleistet, sodass Reste dieser unkonservierten Spüllösungen nach der einmaligen Nutzung verworfen werden sollen. Alternativ sind insbesondere für die ambulante Wundtherapie zunehmend konservierte Wundspüllösungen erhältlich, die nach Anbruch bis zu 8 Wochen verwendet werden können. Der Einsatz von Leitungswasser ist entsprechend den aktuellen Empfehlungen der Kommission für Krankenhaushygiene- und Infektionsbekämpfung (KRINKO) nur dann zulässig, wenn es mit einem Sterilfilter filtriert wird [19]. Im Rahmen des Débridements sollen zudem alle avitalen Bestandteile in den Wunden entfernt werden [20]. So wird bereits ein Großteil der Bakterien in Wunden eliminiert.

Mit dem Buchstaben I werden alle therapeutischen Maßnahmen, die der Infektionskontrolle dienen, beschrieben. Für die antiseptische Wundtherapie stehen verschiedene Behandlungsoptionen als Wundspülung, Gel oder Wundauflage zur Verfügung. Für die meisten Patienten mit chronischen Wunden ist Polihexanid (PHMB) in den verschiedenen Applikationsformen das Mittel der ersten Wahl ([9, 21]; Tab. 7). Auch wenn es heute wenig zytotoxische und effektive Wundantiseptika gibt, rechtfertigt dies deren ungezielten Langzeiteinsatz nicht. Vielmehr soll es zu einem individuellen, gezielten und zeitlich begrenzten Einsatz kommen. Hier wird eine maximale Therapiedauer von 14 Tagen empfohlen [2, 22]. Danach sollte die Indikation erneut kritisch überprüft werden.

**Table 7:** 

Indikation	Antiseptischer Wirkstoff	
	1. Wahl	2. Wahl
Kritisch kolonisierte und infektionsgefährdete Wunden	PHMB	NaOCl/HOCl, Hypochlorit, Silber, OCT/PE
Verbrennungswunden	PHMB	NaOCl/HOCl
Biss‑, Stich‑, Schusswunden	PVP-Jod	OCT/PE
MRE-kolonisierte oder -infizierte Wunden	OCT/PE	OCT, PHMB, Silber
Dekontamination akuter und chronischer Wunden	NaOCl/HOCl, PHMB, Octenidin	OCT/PE
Peritonealspülung	NaOCl/HOCl	–
Risiko der Exposition des ZNS	NaOCl/HOCl	–
Wunden mit fehlender Abflussmöglichkeit	NaOCl/HOCl	–

*PHMB* Polihexanid, *NaOCl* Natriumhypochlorit, *HOCl* hypochlorige Säure, *OCT/PE* Octenidin/Phenoxyethanol, *PVP* Polyvinylpyrrolidon, *MRE* multiresistente Erreger

## Fazit für die Praxis

Lokale Wundinfektionen sind ein multidisziplinäres Problem und können zu zahlreichen Komplikationen, insbesondere bei Patienten mit chronischen Wunden, führen. Daher sollten diese lokalen Wundinfektionen möglichst frühzeitig diagnostiziert und adäquat therapiert werden. Der TILI-Score ermöglicht die rasche Einschätzung im Hinblick auf das Vorliegen einer lokalen Wundinfektion. Mit dem W.A.R.-Score können zudem Patienten mit einem erhöhten Infektionsrisiko identifiziert werden. Therapeutisch kann ein Großteil der Bakterien auf Wunden bereits durch effektive Wundreinigung und/oder Débridement entfernt werden. Für viele Patienten mit chronischen Wunden ist ansonsten Polihexanid (PHMB) das Wundantiseptikum der ersten Wahl. Allerdings sollte die Indikation von Wundantiseptika spätestens nach 14 Tagen kritisch überprüft werden.
